# Potential Risk Areas of *Aedes albopictus* in South-Eastern Iran: A Vector of Dengue Fever, Zika, and Chikungunya

**DOI:** 10.3389/fmicb.2017.01660

**Published:** 2017-09-05

**Authors:** Jalil Nejati, Rubén Bueno-Marí, Francisco Collantes, Ahmad A. Hanafi-Bojd, Hassan Vatandoost, Zabihollah Charrahy, Seyed M. Tabatabaei, Mohammad R. Yaghoobi-Ershadi, Abdolghafar Hasanzehi, Mohammad R. Shirzadi, Seyed H. Moosa-Kazemi, Mohammad M. Sedaghat

**Affiliations:** ^1^Department of Medical Entomology and Vector Control, School of Public Health, Tehran University of Medical Sciences Tehran, Iran; ^2^Departamento de Investigación y Desarrollo (I+D), Laboratorios Lokímica Valencia, Spain; ^3^Department of Zoology and Physical Anthropology, University of Murcia Murcia, Spain; ^4^Department of Environmental Chemical Pollutants and Pesticides, Institute for Environmental Research, Tehran University of Medical Sciences Tehran, Iran; ^5^Department of Natural Resources and Environmental Sciences, Tehran University Tehran, Iran; ^6^Infectious Diseases and Tropical Medicine Research Center, Zahedan University of Medical Sciences Zahedan, Iran; ^7^Zoonoses Control Department, Ministry of Health and Medical Education Tehran, Iran

**Keywords:** *Aedes albopictus*, dengue fever, zika virus, modeling, analytical hierarchy process, geographical information system, remote sensing

## Abstract

The possibility of the rapid and global spread of Zika, chikungunya, yellow fever, and dengue fever by *Aedes albopictus* is well documented and may be facilitated by changes in climate. To avert and manage health risks, climatic and topographic information can be used to model and forecast which areas may be most prone to the establishment of *Ae. albopictus*. We aimed to weigh and prioritize the predictive value of various meteorological and climatic variables on distributions of *Ae. albopictus* in south-eastern Iran using the Analytical Hierarchy Process. Out of eight factors used to predict the presence of *Ae. albopictus*, the highest weighted were land use, followed by temperature, altitude, and precipitation. The inconsistency of this analysis was 0.03 with no missing judgments. The areas predicted to be most at risk of *Ae. albopictus*-borne diseases were mapped using Geographic Information Systems and remote sensing data. Five-year (2011–2015) meteorological data was collected from 11 meteorological stations and other data was acquired from Landsat and Terra satellite images. Southernmost regions were at greatest risk of *Ae. albopictus* colonization as well as more urban sites connected by provincial roads. This is the first study in Iran to determine the regional probability of *Ae. albopictus* establishment. Monitoring and collection of *Ae. albopictus* from the environment confirmed our projections, though on-going field work is necessary to track the spread of this vector of life-threatening disease.

## Introduction

The Asian tiger mosquito, *Aedes albopictus* (Skuse, 1894) (*Diptera*: Culicidae), is known as a competent vector for at least 22 arboviruses, including Zika, dengue fever (DF), and chikungunya (Gratz, 2004; Wong et al., 2013; Collantes et al., 2015). Several studies have warned about the rapid expansion of *Ae. albopictus* around the globe (Roiz et al., 2011; Rochlin et al., 2013; Kraemer et al., 2015). Despite being incapable of flying a distance greater than 800 m, this species has been able to spread from native tropical and subtropical areas of Southeast Asia to America, Europe and Africa as well as to Indo-Pacific and Australian regions in a matter of decades (Roiz et al., 2011; Bueno-Marí and Jiménez-Peydró, 2015; Kraemer et al., 2015). Specimens of *Ae. albopictus* have been observed in southeast Iran (Doosti et al., 2016), which was not unexpected after detecting dengue seropositivity in residents of the south-eastern province of Sistan and Baluchestan (Chinikar et al., 2013). Iran remains at risk of increasing colonization by *Ae. albopictus* from neighboring Pakistan, where there is a history of DF outbreaks (Mukhtar et al., 2011; Rasheed et al., 2013; Khan et al., 2015; Suleman et al., 2016). Chikungunya and Zika have been identified in mosquitoes and sporadically in humans in Pakistan, with positive serological tests signifying widespread population exposure to this virus (CDC, 2016; Kindhauser et al., 2016).

In the late autumn of 2014, *Ae. albopictus* was first reported in the Sistan and Baluchestan Province of Iran in the southeast of the country bordering Pakistan (Doosti et al., 2016). Prior to that, in 2012, the presence of specific IgG, IgM and viral nucleic acid of dengue virus had been detected in the blood of Iranian residents of the province, and were referred to the arboviruses laboratory of the Pasteur Institute of Iran (Chinikar et al., 2013). Again, in 2014, the dengue virus was detected in blood donors in the Chabahar district of the then province (Aghaie et al., 2014).

Dengue fever and, more recently, the Zika virus are considered serious threats to human health due to their increasing abundance and the adaption of their vectors (Banu et al., 2011; Medlock et al., 2012; Petersen et al., 2016). The transmission risk of these viruses depend on the densities of *Ae. albopictus* which, in turn, depend on climatic parameters that control their habitat. Climatic factors can be used to forecast potential establishment areas of *Ae. albopictus* via modeling, which include: mean annual temperature (AnnT^mean^), precipitation, altitude, and relative humidity (RH) (Roiz et al., 2011; Sarfraz et al., 2014; Bueno-Marí and Jiménez-Peydró, 2015; Collantes et al., 2015). One of the most successful techniques in modeling vectors of DF is known as the ‘Analytical Hierarchy Process’ (AHP) (Aziz et al., 2012). AHP and other climate models can be coupled with Geographic Information Systems (GIS) and remote sensing (RS) to predict areas where disease-vectors may establish, as well as for raising awareness of the status of vector-borne diseases in an effective and timely manner (Roiz et al., 2011; Sarfraz et al., 2014). We utilized these models and methods to forecast the proliferation of *Ae. albopictus* and possible dengue/Zika outbreaks in the southeast of Iran.

Managing outbreaks of dengue or Zika virus would pose significant challenges and could strain a health system already involved in the control of arboviral and parasitic diseases such as Crimean-Congo haemorrhagic fever (Izadi et al., 2004; Mostafavi et al., 2013) and malaria (Vatandoost et al., 2011; Hanafi-Bojd et al., 2012; Nejati et al., 2013). The aim of this study was to identify areas of human health risk posed by the colonization of *Ae. albopictus* in south-eastern Iran and lay the groundwork for future monitoring using GIS and RS.

## Materials and Methods

### Study Area

**FIGURE 1:**
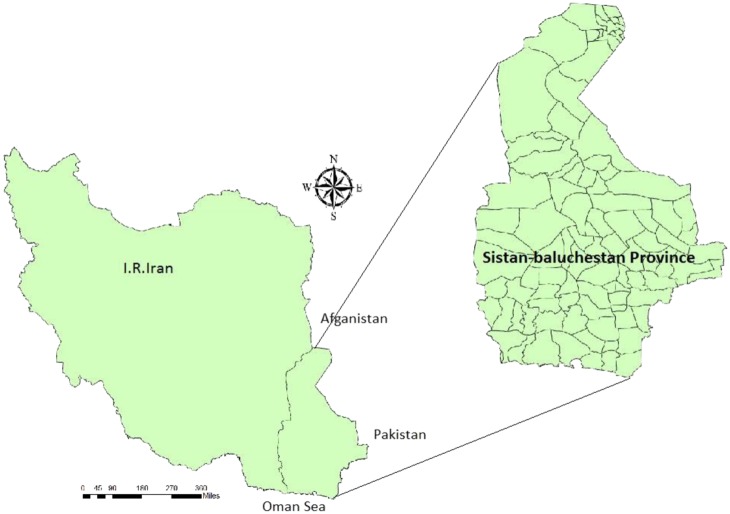
Location of Sistan and Baluchestan Province in the southeast of Iran.

**FIGURE 2:**
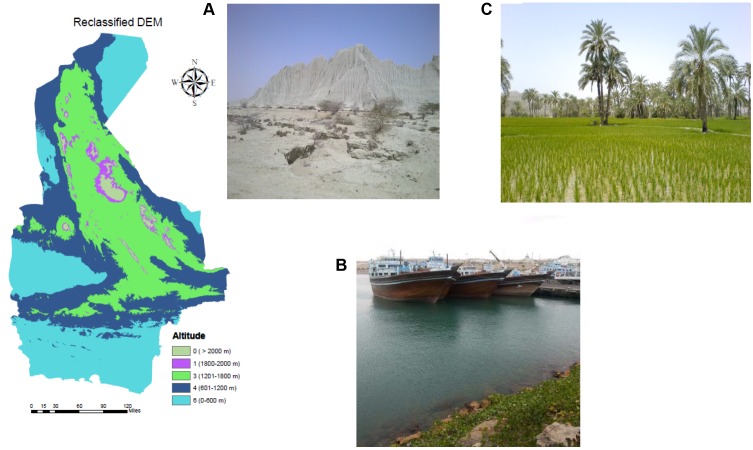
Photos of landforms found in the region of study, including desert **(A)**, a local seaport **(B)**, and a rice and date field **(C)** as well as the topography of the study area.

The study was conducted in the Sistan and Baluchestan province located in southeast Iran, neighboring Pakistan and Afghanistan. It is the largest province in Iran, with an area of 181,785 km^2^, and shares a long border with Pakistan (**Figure 1**). It has a population of approximately 2,534,000 residents in 19 cities, 37 towns, and 9716 villages with an annual growth rate of 1.05% (SCI, 2011)^1^. The climate is generally arid (Amiraslani and Dragovich, 2011) with dust storms and 120-day winds (Alizadeh-Choobari et al., 2014). However, there is substantial climatic diversity due to the dominance of seasonal subtropical high pressures over a large part of the land mass, large internal deserts, the Alpine-Himalayan folded system and the surrounding Jazmurian basin to the west. The influences of the Arabian Sea, to the south, and Hirmand basin, in the north, result in periodic Monsoon systems that can cause a notable ecological phenomenon (SBMO, 2016)^2^. This part of Iran is one of the few with summer rainfall and the main source of humidity stems from the Bengal Gulf streams, coming only in summer (Dinpashoh et al., 2004). Common landscapes in the province are shown in **Figure 2**.

### Identification of Parameters

It was necessary to identify the most important ecological and climatic parameters that affect the survival of *Ae. albopictus* prior to formulating the AHP model. Based on an extensive literature review and consultation with experts on *Ae. albopictus* ecology (Dr. Rubén Bueno-Marí, Dr. Francisco Collantes, and Prof. David Roiz), eight criteria were selected that have the greatest effect on distributions of *Ae. albopictus* populations; these include: precipitation, relative humidity, temperature, land use/anthropization, altitude, wetlands, normalized difference vegetation index (NDVI), soil type, and distance from seaports.

### Analytical Hierarchy Process

#### Formulation of AHP Model

Analytical Hierarchy Process is a structured technique that incorporates both mathematical and psychological components to represent elements of a problem quantitatively in order to facilitate decision-making (Aziz et al., 2012). The priorities and the impact of each element are computed based on the score assigned by the judgments (Saaty, 1990). A pairwise comparison matrix that included the eight aforementioned factors was used to survey 11 scholars and experts who have written authoritative articles on *Ae. albopictus* ecology, and are from Belgium, France, Germany, Iran, Italy, Spain, Sri Lanka, and the United Kingdom. Participants were provided guidelines that asked them to rate the importance of each factor defined by numbers 1–9 or the reciprocal (i.e., 1/2–1/9). The ranking system was as follows: ‘1’ designated equal importance; ‘3’ moderate importance; ‘5’ essential importance; ‘7’ importance; and ‘9’ absolute importance. Even numbers were designated as intermediate values between the two adjacent judgments. The reciprocal was used to demonstrate the relative importance of variables to each other. For example, ‘1/8’ meant that variable ‘x’ is less importantly than ‘y,’ while the reverse (‘8’) meant that ‘x’ was more important. All responses were averaged based on the geometric mean and the weight and priority of each parameter was obtained by Expert Choice 11 software (Ishizaka and Labib, 2009). This software is a powerful decision-making implementation of AHP.

## Data Collection

### Meteorological Parameters

**FIGURE 3:**
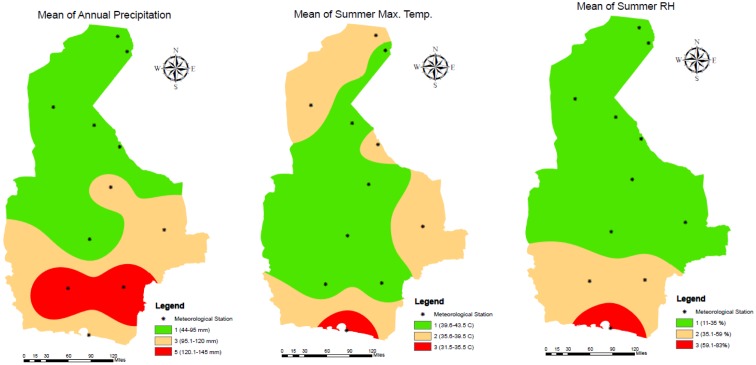
Location of meteorological stations and reclassified maps of temperature, RH and precipitation for the study site, 2011–2015.

Five-year weather data was collected from 11 meteorological stations across the Sistan and Baluchestan province of Iran. These stations were in the following cities/regions: Zabol: 1, Zahak: 2, Zahedan: 3, Mirjaveh: 4, Nosratabad: 5, Khash: 6, Saravan: 7, Iranshahr: 8, Rask: 9, Nikshahr: 10, and Chabahar: 11 (**Figure 3**). Meteorological data was collected over 60 months, from January 2011 to December 2015 and is summarized in **Table 1**. We focused on precipitation, temperature and RH data. Precipitation creates conditions that provide breeding sites and a moist microclimate for adult mosquitoes (Sarfraz et al., 2014). Annual, spring, summer, autumn, and winter precipitation values were not significantly correlated to each other (Correlations test; *P* > 0.05). Pluviometric conditions with annual rainfall between 200 and 500 mm have been reported in established areas (Bueno-Marí and Jiménez-Peydró, 2015). Although studies have shown a correlation between seasonal rainfall and *Ae. albopictus* density, differing views exist in the literature. This species can propagate in some rainfall-independent breeding sites because of its behavior as a container-breeding mosquito (Waldock et al., 2013). Due to the dry climate and low rainfall of the province, we opted to use annual precipitation (AnnP) based on the average precipitation at each meteorological station.

**Table 1:** Meteorological data from all weather stations collected from 2011 to 2015.

Station No.	Climate^∗^	Temperature average within 5-year study (°C)		Precipitation average within 5-year study (mm)		RH average within 5-year study		Annual average of days < 0°C^∗^	Average annual of days < -5°C	Average annual of days > 35°C^∗^
							
		Mean ± STDEV	Confidence interval 95%	Mean ± STDEV	Confidence interval 95%	Mean ± STDEV	Confidence interval 95%			
1	Hot and dry	23.2 ± 1.3	20.6-25.7	44.38 ± 17.4	36.6-52.1	30.8 ± 1.8	27.3-34.5	17	0	155
2	Hot and dry	23.1 ± 1.2	20.7-25.6	57.82 ± 32.1	43.4-72.2	30.1 ± 1.8	26.5-33.7	13	0	165
3	Semi-arid and warm/temperate	19.1 ± 1.0	17.1-21.2	75.42 ± 29.43	62.3-88.6	30.1 ± 1.6	26.9-33.4	43	0	90
4	–	23.9 ± 1.1	21.6-26.1	54.6 ± 37.7	37.8-71.5	22.5 ± 1.5	19.6-25.4	–	0	–
5	–	22.5 ± 1.2	20.0-24.9	87.9 ± 45.5	67.5-108.2	25.2 ± 1.7	21.6-28.7	–	0	–
6	Semi-arid and warm/temperate	20.4 ± 1.0	18.4-22.5	106.0 ± 73.3	73.3-138.8	28.1 ± 1.5	25.0-31.2	20	0	110
7	Semi-arid and warm/temperate	22.3 ± 1.1	20.2-24.3	103.9 ± 56.9	78.3-129.3	28.8 ± 1.1	26.6-31.1	10	0	139
8	Hot and dry	27.2 ± 1.1	25.1-29.4	64.9 ± 42.1	46.1-83.8	26.2 ± 1.1	23.9-28.5	1	0	187
9	Hot	28.5 ± 0.8	26.9-30.1	145.6 ± 89.1	105.7-185.4	38.7 ± 1.4	35.8-41.6	0	0	24
10	Hot	27.9 ± 0.8	26.2-29.6	142.0 ± 39.4	124.4-159.6	38.8 ± 1.2	36.6-40.9	0	0	231
11	Costal	26.5 ± 0.5	25.6-27.5	106.7 ± 50.7	84-129.4	71.9 ± 1.5	68.8-74.9	0	0	13


*^∗^Based on the annual report of the provincial meteorological organization (SBMO, 2016)^2^.*

*Aedes albopictus* cannot typically survive average temperatures less than -5°C or greater than +41°C, though snow cover, in winter, and shade, in summer, can mitigate temperature and provide refugia (Brady et al., 2013). A mean annual temperature (AnnT^mean^) of 11°C is also considered as a critical temperature for cessation of *Ae. albopictus* life cycle (Roiz et al., 2011). A variety of temperature calculations were assessed, including the monthly temperature, mean of maximum summer temperature (MxST), mean of minimum winter temperature (MiWT), mean of winter temperature (MWT), and temperature in January. A significant correlation was found between MxST, MiWT, and MWT (*P* < 0.05). Given the arid climate in our study area, we selected MxST as a major constraint in our model.

Conditions of high temperature and low RH have deleterious effects on adult populations (Alto and Juliano, 2001). Humidity also has a direct effect on the durability of breeding sites and larval survival rates, where high RH (68–80%) can increase fecundity (Sarfraz et al., 2014). Among diapause eggs, *Ae. albopictus* can survive the longest at low RH (44%) compared to *Stegomyia* strains (Sota and Mogi, 1992). RH values for annual, spring, summer, autumn, and winter were analyzed and all showed significant pairwise correlation. Without a real difference between each variable, we utilized the mean summer RH (MSR) at each station as a limited factor for modeling.

### Remote Sensing Parameters and Image Pre-processing

Images from Landsat and Terra satellites were used to calculate the Digital Elevation Model (DEM), water bodies, urban/rural (residential) areas and NDVI. For all but DEM, calculations were based on 16 images from the Landsat 8 satellite, each covering an area of 185 km^2^.

A seamless coverage of the study area was prepared by tiling satellite images that had been color balanced and mosaicked using ENVI V.5.0 (EXELIS Visual Information Solutions, Boulder, CO, United States). Residential areas were classified using visual interpretation on virtual color combination (*RGB* = bands 7, 5, and 2). DEM was calculated from 3N and 3B bands taken from the ASTER (Advanced Space-borne Thermal Emission and Reflection Radiometer) sensor on the Terra satellite with a nominal resolution of 25–30 meters for each pixel size. The normalized index of different water (NDWI) was used to calculate the water bodies’ parameter. The index was defined as follows: (B2-B7)/(B2+B7), where values greater than 0.2 were considered as bodies of water. NDVI was generated using red and near-infrared bands of Landsat 8 with the following equation: (B5-B4)/(B5+B4).

### Classification and Valuation of Factors

Prior to GIS mapping, factors were classified based on literature reviews. To ensure the quality of the classification, we received confirmation from three international scholars (Dr. Rubén Bueno-Marí, Dr. Francisco Collantes, and Prof. David Roiz). Precipitation ranged from 44 to 146 mm and was grouped into three classes: 44–95 mm, 95.1–120 mm, and 120.1–146 mm and attributed three values: 1, 3, and 5. The summer maximum average temperature ranged from 31.5 to 43.5°C and was also grouped into three classes with descending values 3, 2, and 1, with lower values allocated to the high temperatures to signify a deterrent role in adult mosquito survival. The classification maps based on inverse distance weighted (IDW) interpolation of temperature, RH and precipitation are presented in **Figure 2**.

Colonies of *Ae. albopictus* are commonly reported in urban and suburban areas (Devi and Jauhari, 2004; Roiz et al., 2011; Bueno-Marí and Jiménez-Peydró, 2015; Collantes et al., 2015; Dhimal et al., 2015). *Ae. albopictus* is a weak flier with a flight range of less than 1 km (Rochlin et al., 2013). The unaided flight range of this species has been reported to be between 100 and 800 m with the average falling in the range of 100–200 m followed by 200–400 m (Honório et al., 2003; Nihei et al., 2014). Based on these results, we drew concentric circles and hazard bands (buffer) up to 800 m via straight-line distance around any polygons, ploy lines and points of cities, villages, roads, costumes, international airports, railway stations, and sea/ground entrance ports. The land use/anthropization parameter was then grouped into six classes: 0–100 m, 101–200 m, 201–400 m, 401–600 m, 601–800 m, > 800 m and assigned descending values: 6, 5, 3, 2, 1, and 0, respectively.

Altitude was considered as a limitation to the dispersal of *Ae. albopictus* with the threshold set at 2100 m (Dhimal et al., 2015) and greatest expected density of survivorship at 300–600 m (Devi and Jauhari, 2004). Elevation ranged between 0 and 3912 m for the study area and was grouped into five classes and values including 0–600 m (6), 601–1200 m (4), 1201–1800 m (3), 1801–2000 m (1) and > 2000 m (0). The classification map of altitude is shown in **Figure 2**.

Water bodies were attributed a 200 m buffer and classified into two classes: pixels with (2) and without wetlands (1). The vegetation or land cover was used to assign potential breeding habitats (Sarfraz et al., 2014). NDVI ranged from -1 to 1 and was grouped into five classes: -1 to 0 indicated areas without vegetation; 0 to 0.25 indicated low vegetation; 0.25 to 0.50 indicated moderate vegetation; 0.50 to 0.75 indicated high vegetation and 0.75 to 1 designated densely vegetated (or ‘very high’), with a value of 6 assigned to areas of very high vegetation and a value of 0 to areas without vegetation.

The ‘distance from border’ was calculated the same way as land use/anthropization and was grouped into six classes with descending values. The scale, classes and values of each criterion are shown in **Table 2**.

**Table 2:** The scale, classes, and values of the parameters used in Geographic Information Systems (GIS) mapping.

Criteria	Scale	Min–max in the study area	Classification and value					
			
			Class 1 (value)	Class 2 (value)	Class 3 (value)	Class 4 (value)	Class 5 (value)	Class 6 (value)
Precipitation	Average of annual precipitation 2011–2015	44–146 mm	44–95 (1)	95.1–120 (3)	120.1–146 (5)	–	–	–
Relative Humidity (RH)	Average of summer RH 2011–2015	11–83%	11–35% (1)	35.1–59% (2)	59.1–83% (3)	–	–	–
Temperature	Average of maximum summer Temperature 2011–2015	31.5–43.5°C	31.5–35.5 (3)	35.5–39.5 (2)	39.5–43.5 (1)	–	–	–
Land use/Anthropization	Villages+Cities	–	0–100 (6)	100–200 (5)	200–400 (3)	400–600 (2)	600–800 (1)	> 800 (0)
Altitudes	Elevation	0–3912 m	0–600 (6)	600.1–1200 (4)	1200.1–1800 (3)	1800.1–2000 (1)	> 2000 (0)	–
Wetlands	Surface water bodies	^∗^200 mbuffer	Wetland (1)	No wetland (0)	–	–	–	–
NDVI	NDVI ^∗^100 m buffer	-1–1	-1–0 (1)	0–0.15 (3)	0.15–0.50 (4)	0.50–0.75 (5)	> 0.75 (6)	–
Distance from Border	Sea ports + Entrance points + Customs +Int. airports + Int. rail station		0–100 (6)	100–200 (5)	200–400 (3)	400–600 (2)	600–800 (1)	> 800 (0)


### GIS Analysis

**FIGURE 4:**
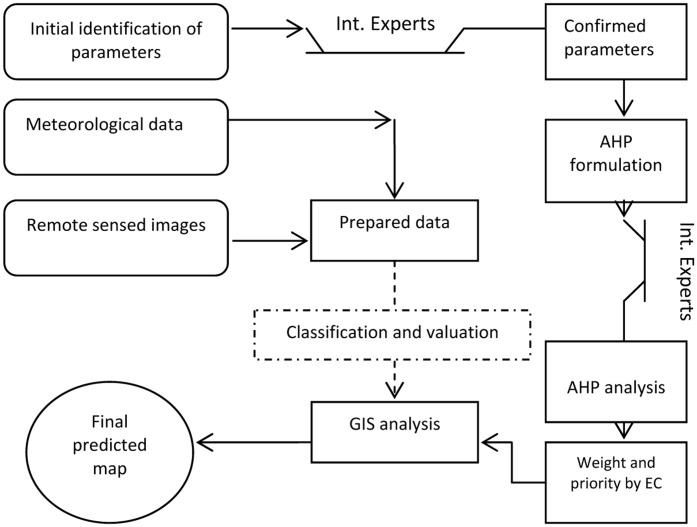
The modeling framework for estimating areas with the potential presence of *Aedes albopictus* based on Analytical Hierarchy Process (AHP), remote sensing (RS), and Geographic Information Systems (GIS).

Each of the eight factors were added as a layer in ESRI ArcGIS 10.3 software. The IDW was used to interpolate the boundaries between meteorological stations for precipitation, RH and temperature. They were reclassified based on previous classification and valuation. The reclassification was also applied for other layers. Before reclassifying, the straight-line approach was implemented for buffering around cities, villages, seaports, entry points, international airports, and railway station. **Figures 2**, **3** show the reclassified map of altitude and meteorological data. Although the distance from a border was conflated with distance from land and sea in certain situations, we considered all seaports, each entry point to the province from Pakistan (ranging from the gravel roads to the paved and asphalted), international airports, costumes, and international railway station for preparation of this layer via punctuation in Google Earth and transferring to the GIS. The final predictive model exhibiting areas with the greatest potential for proliferation of *Ae. albopictus* was developed using Spatial Analyst of ArcMap. The weight of each parameter (layer), previously obtained by AHP, was used for raster calculation. A summary of our methodology is provided in **Figure 4**.

## Results

### AHP; Criteria Weighting and Priority by EC

**FIGURE 5:**
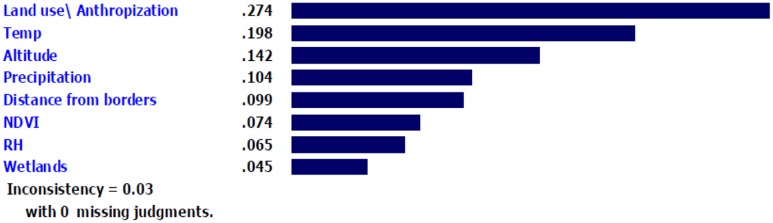
The weight and priority of model parameters with respect to predicting the potential presence of *Ae. albopictus*.

After weighing the eight criteria by AHP, the factor with the greatest weight (0.274) and priority was land use/anthropization for assessing the presence of *Ae. albopictus*. The next best criteria were temperature, altitude and precipitation, while proximity to wetland had the lowest weight (0.042) and priority (**Figure 5**). The inconsistency of this analysis was 0.03 with no missing judgments.

### Predictive Modeling

#### Potential Habitable Regions of *Ae. albopictus*

**FIGURE 6:**
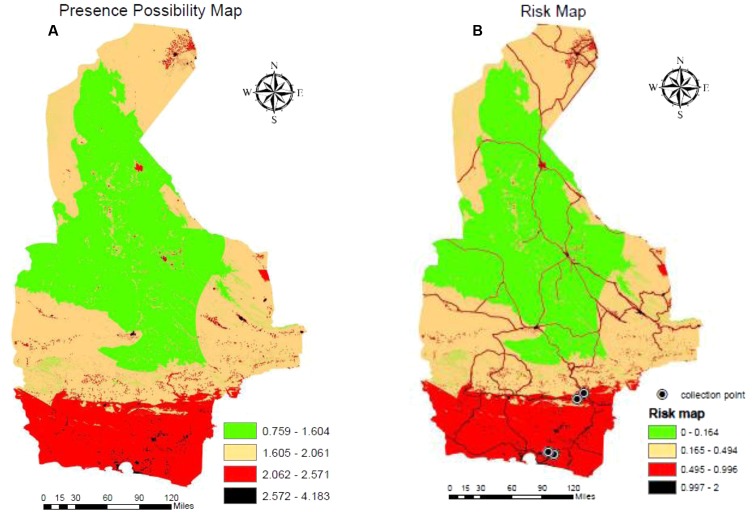
GIS mapping of the potential presence of *Ae. albopictus* **(A)** and a map of risk of exposure **(B)** that includes locations where specimens of *Ae. albopictus* have been recovered.

Areas where *Ae. albopictus* has the potential to be present are shown in **Figure 6** based on the representation of the AHP model. The Natural Breaks (Jenks) classification method divided the potential presence of this species into four classes: 0.759–1.605, 1.606–2.061, 2.062–2.572, and 2.573–4.1834. Classes 3 and 4, where values are greater than > 2.06, can be considered areas with potential of presence for *Ae. albopictus*. The map shows that southern areas with a coastal climate are most suitable for supporting populations of *Ae. albopictus*.

#### Map of Risk Areas

To identify areas with greatest potential risk, we multiplied the estimated presence of *Ae. albopictus* by the main means of dispersal. Therefore, we added the reclassified layer of provincial roads with a maximum 800 m buffer via Euclidian Direction. This map shows the risk areas and most likely habitable regions for *Ae. albopictus*. Again, the potential of presence of this species was classified into four classes with values 0–2 based on the Natural Breaks classification method. Classes 3 and 4 with values > 0.49 show the moderate and high-risk areas with potential of presence for *Ae. albopictus* (**Figure 6**). The majority of risk areas are found in the southern part of the province, which include 834 villages. Doosti et al. (2016) previously reported the detection of *Ae. albopictus* in four of these villages (Vashname dorri, Paroomi, Lashar, and Rask; noted in **Figure 6**), confirming the validity of our model and the provided map. Also, some bioclimatic variables and altitudes of the collection points of *Ae. albopictus* are presented in **Table 3**.

**Table 3:** Bioclimatic variables at collection points of *Aedes albopictus*.

Variable	Min	Max	Mean ± *SD*	Confidence interval 95%
Altitude	8	427	280 ± 235.8	143.9–416.1
Annual precipitation^∗^	106.7	145.6	131.4 ± 21.5	119–143.8
Winter precipitation^∗^	38	62.8	51.6 ± 12.6	44.3–58.9
Annual Temperature^∗^	26.6	28.5	27.6 ± 1.0	27.0–28.2
Winter Temperature^∗^	19.5	21.5	20.5 ± 1.0	20.0–21.1
Summer Temperature^∗^	29.8	33.6	32.5 ± 2.4	31.1–33.9
Annual RH^∗^	38.7	71.8	49.8 ± 19.1	38.7–60.8
Winter RH^∗^	38.1	60.5	59.8 ± 20.9	47.8–71.9
Summer RH^∗^	43.9	83.4	48.8 ± 49.0	47.7–57.6


*^∗^Average of the variable during 2011–2015.*

## Discussion

This is the first study to model areas in Iran at greatest risk for colonization by disease-vectors *Ae. albopictus*. Our model’s predictions are in agreement with the most recent field reports, demonstrating the utility of our risk map for forecasting the spread of *Ae. albopictus* and for monitoring and ensuring preparedness for potential DF or Zika outbreaks. To date, only dengue has been detected in Iran (Chinikar et al., 2013), but in neighboring Pakistan, dengue, Chikungunya fever, and sporadic human cases of Zika have been reported along with the detection of infected mosquitoes (Kindhauser et al., 2016). These findings clearly indicate the risk posed by the colonization of south-eastern parts of Iran by *Ae. albopictus*.

*Aedes albopictus* is not the primary vector of Chikungunya and dengue viruses (Waldock et al., 2013; Collantes et al., 2015; ECDC, 2016) and has a lesser role in the transmission of Zika compared to *Ae. aegypti* (Chouin-Carneiro et al., 2016; Jupille et al., 2016). Yet, the competence of *Ae. albopictus* for Zika transmission has been proven in a laboratory setting (Di Luca et al., 2016; WHO, 2016) and, recently, the African lineage of Zika has been isolated from *Ae. albopictus* in Gabon (Grard et al., 2014). Though there has yet to be a case of *Ae. albopictus* transmission of Zika, the risk should not be ignored as the virus has been isolated in its salivary glands, suggesting a strong likelihood of it acting as a vector for Zika (Ioos et al., 2014).

The selection of valid parameters has an important role in developing a reliable model and can be achieved through knowledge of mosquito ecology (Waldock et al., 2013). We were able to select factors through an extensive literature review and the participation of international scholars. The most heavily weighted parameters identified in our model have been previously used in modeling studies, such as land use, land cover, temperature, precipitation, RH, and altitude (Aziz et al., 2012; Rochlin et al., 2013; Sarfraz et al., 2014).

Our finding that land use/anthropization had the highest weight and priority, followed by temperature, altitude, and precipitation, paralleled the findings from a previous study that used AHP to model dengue hotspots in Malaysia (Aziz et al., 2012). Similarly, land use was determined as the second most important variable in a modeling study in the north-eastern United States. Hotspots for *Ae. albopictus* were more urban and suburban due to the affinity of this species to urbanized environments (Rochlin et al., 2013). Villages, too, are likely habitats (Ganushkina et al., 2016) and were where the first collection of this species in Iran occurred. Therefore, we calculated villages similar to cities in our model.

Despite parallels to previous work, our identification and selection of some parameters was different. Most papers have constrained *Ae. albopictus* fitness using temperatures in January, whereas we utilized the average summer maxima (Takumi et al., 2009; Neteler et al., 2011; Caminade et al., 2012). The temperature in January would be a stronger limiting factor in the Northern hemisphere (Waldock et al., 2013). Due to the dominance of hot and dry climates in our study area, overwintering was easier than escape from summer heat given that minimum temperature for biological activity of *Aedes* mosquitoes is 6–10°C (Dhimal et al., 2015) and temperatures do not drop below 5.5°C in our region of study. On the other hand, the average annual of glacial days (days when the temperature drops below freezing) in each station was rare, while days with a temperature greater than 35°C were common. At temperatures > 40°C, the survival of immature and adult stages of *Ae. albopictus* are negatively impacted, but these conditions are less documented (Waldock et al., 2013). In this region of Iran, it is possible that summer temperatures are more important for modeling because the most suitable temperature range for reduction of extrinsic Dengue incubation is between 32 and 35°C (Alto and Juliano, 2001).

Relative humidity is considered an effective predictive measure of population dynamics and egg, larval and adult stages of *Ae. albopictus* (Alto and Juliano, 2001; Waldock et al., 2013). This factor was selected in much the same way as other studies, but, again, with a slight difference. In contrast with other studies that have applied annual RH (Almeida et al., 2005), we used the average RH in summer. In our case, hot and dry regimes with low RH in the summer are likely to be a stronger limiting factor due to reduction in adult reproduction (Alto and Juliano, 2001). Yet, due to the important role of high RH in increasing abundances (Waldock et al., 2013; Sarfraz et al., 2014), we allocated greater values to high RH in the classification phase. Similar to low RH, annual rainfall plays an important part in most modeling (Aranda et al., 2006; Dhimal et al., 2015) as a limited factor (Medlock et al., 2006). The importance of this parameter grows under drought conditions when aquatic habitats dry up (Alto and Juliano, 2001). Thus, we weighted our criteria (i.e., decussate values 1, 3, and 5 were chosen for low precipitation to high) since Sistan and Baluchestan province has experienced drought in recent years (Abbaspour and Sabetraftar, 2005; Lashkaripour and Zivdar, 2005).

Apart from meteorological parameters, certain landforms can play an important role in the presence of *Ae. albopictus* (Rochlin et al., 2013). The inclusion of wetlands in our model did not have a significant predictive value. This is consistent with the fact that *Ae. albopictus* is considered a peridomestic mosquito and is known as a container-breeding mosquito that prefers rain-filled containers (Vanwambeke et al., 2011; Dhimal et al., 2015). Wetlands can provide a natural reservoir that can enable it to spread quickly (Dale and Knight, 2008) and small numbers of *Ae. albopictus* have been collected from the wetlands of Valencian autonomous regions (Bernues Baneres et al., 2012). Also in Iran some of larvae of this species were collected from natural breeding sites (Doosti et al., 2016). Thus, we included wetlands in our model, but assigned a low value (1) commensurate with the small role of wetlands in its ecology.

The effects of global warming on climate and landforms is commonly argued as the major reason for increased vector-borne diseases. Yet, road construction and urban development have had significant impacts on the spread of these diseases. There is evidence for the occasional transport of *Ae. albopictus* by car and/or trucks in Europe through artificial ovipositor sites such as tires and flowers (Takken and Knols, 2007; Collantes et al., 2015). In our study, roads served as a vector habitat developer layer to obtain the risk map. Roads were reported as important means of dispersal of this species in Vietnam (Higa et al., 2010). This important caused that in a research on the effect of climatic factors driving invasion of *Ae. albopictus* in Northern Italy, some of sampling stations were positioned along the roads as passive transport way of this species (Roiz et al., 2011). We feel that the most innovative models will address the role of roads as contributing factors to the risk of *Ae. albopictus* and believe this research sets an important precedent. In the final risk map, the collection points, reported in a previous research (Doosti et al., 2016), were close to the roads that can confirm this equation.

Among various classification techniques supplied by mapping software, the Natural Breaks method can find cut points for class creation based on the data distribution. There is no specific rule about the number of classes, but its acceptable range is between 4 to 11 classes (Werneck, 2008); in our case it produced 4 classes. Natural Breaks was used for identification of malaria hotspots in India (Srivastava et al., 2009) and was suggested to be the best method for predicting the most severe threat of dengue in a case study in Cambodia (Owen and Slaymaker, 2005). The Equal Intervals as another common classification method might be a good choice in rectangular data distribution (Werneck, 2008). This method has been rather useful for finding areas with high probability of presence of cutaneous Leishmaniasis vectors (Hanafi-Bojd et al., 2015). Our research provides an example of the effective use of Natural Breaks for future modeling work.

*Aedes albopictus* is considered as a dangerous species that is highly adaptive and present in both tropical and temperate climates (Roiz et al., 2011). In our study, the most suitable areas for colonization, where specimens had previously been collected (Doosti et al., 2016), were in coastal climates with low elevation. The presence of *Ae. albopictus* in lowlands with an altitude of less than 100 m was demonstrated in a region in Nepal with comparable climate (Dhimal et al., 2015). The low rainfall in northern parts of our study area likely limit conditions for establishment of *Ae. albopictus*. In a study in the United Kingdom, annual rainfall of less than 300 mm limited survival. The mean AnnP reported for our region in Iran is well below the 500 mm annual rainfall reported to be sufficient for establishing populations (Medlock et al., 2006). Yet, specimens have been collected from comparable climates in India, where sparse vegetation and water stored in human-made containers (Roiz et al., 2011; Sarfraz et al., 2014) supported populations similar to what was seen in the south-eastern area of our study site.

For the first time, meteorological, topography, and climatology variables were applied to predict areas in Iran that may support populations of *Ae. albopictus*. We demonstrated the innovative use of AHP and the importance of adding roads during prediction mapping. We recommend further improving RS capabilities as collecting meteorological and climatic data from ground stations is time consuming (Roiz et al., 2011; Sarfraz et al., 2014). We also believe long-term data collection and integration with climate change models is an important next step for refining our model. And, although our model was validated by limited field measurements, further monitoring and refinement will be necessary, as well as expanding to more northern provinces in Iran where the populations of *Ae. albopictus* have been predicted (WHO, 2014; Kraemer et al., 2015). The next advancement will be to model actual fluctuations in the abundances of populations.

## Ethics Statement

This sudy has been approved by ethical commitee, Research Deputy, Tehran University of Medical Sciences with the letter-number: IR.TUMS.SPH.REC.1395.507.

## Author Contributions

Conception or design of the work: JN, AH-B, HV, and FC. Data collection: JN, AH, MRS, and SM-K. Data analysis and interpretation: ZC, ST, and MMS. Drafting of the article: JN, AH-B, and HV. Critical revision of the article: RB-M and MY-E. All authors read and approved the final version of the manuscript.

## Conflict of Interest Statement

The authors declare that the research was conducted in the absence of any commercial or financial relationships that could be construed as a potential conflict of interest.

## Footnotes

1 https://www.amar.org.ir/Portals/1/Iran/90.pdf

2 http://www.sbmet.ir/userfiles/uploads/climatesb.pdf
